# Association of TLR 9 gene polymorphisms with remission in patients with rheumatoid arthritis receiving TNF-α inhibitors and development of machine learning models

**DOI:** 10.1038/s41598-021-99625-x

**Published:** 2021-10-11

**Authors:** Woorim Kim, Tae Hyeok Kim, Soo Jin Oh, Hyun Jeong Kim, Joo Hee Kim, Hyoun-Ah Kim, Ju-Yang Jung, In Ah Choi, Kyung Eun Lee

**Affiliations:** 1 College of Pharmacy, Chungbuk National University, 660-1 Yeonje-ri, Osong-eup, Heungdeok-gu, Cheongju, 28160 Republic of Korea; 2 College of Pharmacy, Ajou University, 164 Worldcup-ro, Yeongtong-gu, Suwon, 16499 Republic of Korea; 3 Department of Rheumatology, Ajou University School of Medicine, 164 Worldcup-ro, Yeongtong-gu, Suwon, 16499 Republic of Korea; 4 Division of Rheumatology, Department of Internal Medicine, Chungbuk National University Hospital, 776, 1sunhwan-ro, Seowon-gu, Cheongju, 28644 Republic of Korea

**Keywords:** Rheumatoid arthritis, Personalized medicine

## Abstract

Toll-like receptor (TLR)-4 and TLR9 are known to play important roles in the immune system, and several studies have shown their association with the development of rheumatoid arthritis (RA) and regulation of tumor necrosis factor alpha (TNF-α). However, studies that investigate the association between TLR4 or TLR9 gene polymorphisms and remission of the disease in RA patients taking TNF-α inhibitors have yet to be conducted. In this context, this study was designed to investigate the effects of polymorphisms in TLR4 and TLR9 on response to TNF-α inhibitors and to train various models using machine learning approaches to predict remission. A total of six single nucleotide polymorphisms (SNPs) were investigated. Logistic regression analysis was used to investigate the association between genetic polymorphisms and response to treatment. Various machine learning methods were utilized for prediction of remission. After adjusting for covariates, the rate of remission of T-allele carriers of TLR9 rs352139 was about 5 times that of the CC-genotype carriers (95% confidence interval (CI) 1.325–19.231, p = 0.018). Among machine learning algorithms, multivariate logistic regression and elastic net showed the best prediction with the area under the receiver-operating curve (AUROC) value of 0.71 (95% CI 0.597–0.823 for both models). This study showed an association between a TLR9 polymorphism (rs352139) and treatment response in RA patients receiving TNF-α inhibitors. Moreover, this study utilized various machine learning methods for prediction, among which the elastic net provided the best model for remission prediction.

## Introduction

Rheumatoid arthritis (RA) is a severe chronic inflammatory reaction that occurs in the synovium of joints. Mortality hazards are 60%–70% higher in patients with RA than in those without the disease^1^. Although the exact etiology of RA is still under investigation, several genetic studies have suggested a role of genetic factors^2, 3^. The most well-known genetic risk factors for RA are variations in human leukocyte antigen (HLA) genes, especially the HLA-DRB1 gene^4^. However, many other genes with potential links to RA remain to be investigated in order to discover further genetic risk factors and therapeutic variations for RA.

Tumor necrosis factor alpha (TNF-α) inhibitors play important roles in inflammatory states, including RA^5^. There are five TNF-α inhibitors available for RA treatment (adalimumab, certolizumab, etanercept, golimumab, and infliximab), and clinical efficacies in RA are known to be similar among these agents^6^. Patients with advanced RA are treated with TNF-α inhibitors; however, the efficacy of these treatments is still questionable as several studies have reported that only one-third of the patients benefit from the treatment^7, 8^.

Toll-like receptors (TLRs) play vital roles in both innate and acquired immune systems^9^, and several studies have shown their association with the development of RA^10–12^. Notably, TLRs are known as inducers of TNF-α transcription^13^. Triad3A is an E3 ubiquitin–protein ligase that induces degradation of TLR4 and TLR9^14^. Hence, reduction in endogenous Triad3A results in TLR activation. Since Triad3A acts specifically on TLR4 and TLR9 among the 13 members of the TLR family, the genes encoding TLR4 and TLR9 are important for understanding RA pathogenesis and potential therapeutic intervention^15–18^. A study showed that TLR4 is specifically required for production of osteoclastogenic cytokines, thus, involved in pathophysiology of RA^19^. Moreover, an in vitro study reported that TLR4 is required for the TNF-α expression^20^. Another study revealed that TLR9 level was elevated on circulating and synovial monocyte subsets of RA patients^21^.

Nuclear factor-kappaB (NFkB) is associated with the response to TNF-α inhibitors in autoimmune diseases^22^. Due to this association, several proteins activating NFkB have been discovered and investigated, including TLRs. As TLRs activate pro-inflammatory cytokines including TNF-α and transcription factors such as NFkB, their polymorphisms may potentially affect treatment outcomes^23^.

Recently, machine learning methods have been utilized as tools for decision making and clinical predictions. Compared to traditional predictive models that use selective variables for calculation, machine learning approaches are favorable when developing novel prediction models. Moreover, remission in RA is important since clinical remission is considered a treat-to target goal. Therefore, this study was designed to investigate the effects of polymorphisms in TLR4 and TLR9 on response to TNF-α inhibitor and by training predictive models utilizing various machine learning approaches for remission.

## Methods

### Study patients

This prospective observational two-center study enrolled 105 patients who were prescribed TNF-α inhibitors (adalimumab, etanercept, golimumab, or infliximab) at Ajou University Hospital and Chungbuk National University Hospital between July 2017 and December 2019. Data collection was conducted using electronic medical records. Data on sex, age, weight, height, duration of RA, autoantibodies against rheumatoid factor, anti-cyclic citrullinated peptide, concomitant medications, and comorbidities were collected from electronic medical records. Additionally, baseline data on disease activity score (DAS)-28 and its subcomponents, which included tender joint count (TJC)-28, swollen joint count (SJC)-28, global health (GH), and erythrocyte sedimentation rate (ESR) or C-reactive protein levels, were collected.

A good clinical response to anti-TNF therapy was defined as the basis of the DAS-28 scores. Patients with a DAS-28 score of less than 2.6 after 6 months of TNF-α inhibitor therapy, were considered to be in remission^24^. DAS-28 was calculated as 0.56 × √(TJC28) + 0.28 × √(SJC28) + 0.70 × ln(ESR) + 0.014 × GH^24^.

This study was approved by the Institutional Review Boards of the Ajou University Hospital (approval number: AJIRB-BMR-OBS-17-153) and Chungbuk National University Hospital (approval number: 2017-06-011-004). All patients submitted written informed consents for participation. This study was conducted according to the principles of the Declaration of Helsinki (2013).

### Genotyping methods

To select single nucleotide polymorphisms (SNPs) of TLR4 and TLR9 that might be associated with RA remission, genetic information on TLR4 and TLR9 was obtained from the PharmGKB database, Haploreg 4.1, the NCBI Database of SNPs (dbSNP), and previous studies^22, 25–29^. A total of six SNPS, including four SNPs of TLR4 (rs11536889, rs1927907, rs1927911, and rs2149356) and two SNPs of TLR9 (rs352139 and rs352140), were selected. Tag SNPs were chosen with minor allele frequency (MAF) of ≥ 25% in Japanese and Han Chinese populations using Haploview 4.2. Among selected SNPs, TLR4 SNP rs1927907 and rs1927911 and TLR9 SNP rs352139 were previously studied for autoimmune related conditions^19, 25, 26^.

Genomic DNA of the patients was isolated from ethylenediaminetetraacetic acid (EDTA)–blood samples using the QIAamp DNA Blood Mini Kit (Qiagen GmbH, Hilden, Germany) according to the manufacturer’s protocol. Genotyping was performed using a single-base primer extension assay with TaqMan genotyping assay in a real-time PCR system (ABI 7300, ABI), according to the manufacturer’s recommendations (Supplementary section).

### Statistical analysis and machine learning methods

Student’s *t*-test was used to compare continuous variables between patients who showed good clinical response (remission) and those who did not. Chi-square test or Fisher’s exact test was used to compare categorical variables between the two groups. Multivariable logistic regression analysis was used to examine independent factors affecting remission; factors with a *p-*value less than 0.05 in univariate analysis along with clinically relevant confounders were included in multivariable analysis. The Hosmer–Lemeshow test was performed to confirm the model’s goodness of fit.

This study employed a random forest–based classification approach to analyze the importance of different variables for factors that affect remission. To prevent over-fitting, we selected seven features that are most important. Various machine learning methods such as multivariate logistic regression, elastic net, random forest, and support vector machine (SVM) were utilized for prediction of remission. All the methods were implemented with the caret R package (version 6.0-88, https://github.com/topepo/caret/). The area under the receiver-operating curve (AUROC), to assess the ability of the risk factor to predict complication, and its 95% confidence interval (CI) of each machine learning prediction models were described in this study. A *p*-value of less than 0.05 was considered statistically significant. Univariate statistical analysis was conducted using IBM SPSS statistics, version 20 software (International Business Machines Corp., New York, USA). All other analyses were performed using R software version 3.6.0 (R Foundation for Statistical Computing, Vienna, Austria).

To measure performance of each machine learning model, internal validation was done. The dataset was randomly divided for model development and evaluation in prediction process. After partitioning one data sample into five subsets, one subset was selected for model validation while the remaining subsets were used to establish machine learning models. Each five-fold cross-validation iteration was repeated 100 times to evaluate the power of the machine learning models.

## Results

Among the 105 patients enrolled in this study, 7 patients were excluded due to incomplete medical data. The data from 98 patients receiving TNF-α inhibitors were analyzed. The mean age of the included patients was 53 years (range: 20–82 years), and there were 79 (80.6%) females. The mean duration of RA was 9 years, and 29 patients reached remission. To determine the possible effect of disease status on response to TNF-α inhibitors, baseline DAS-28 and its subcomponents were examined. Baseline DAS-28 and its subcomponents were not statistically significant between the remission and non-remission groups (Table 1). Marginal significance was found according to sex (*p* = 0.059) and hypertension (*p* = 0.060).

**Table 1:** Patient characteristics according to the response at 6 months treatment of TNF inhibitors.

Characteristics, n (%)	Remission (n = 29)	No remission (n = 69)	*p-*value
**Sex**			0.059
Male	9 (31.0)	10 (14.5)	
Female	20 (69.0)	59 (85.5)	
**Age**	51.4 ± 13.4	53.6 ± 13.9	0.399
< 65	26 (89.7)	53 (76.8)	0.142
≥ 65	3 (10.3)	16 (23.2)	
**BMI, kg/m**^**2**^	23.4 ± 3.0	22.3 ± 3.8	0.220
< 23	13 (44.8)	42 (60.9)	0.144
≥ 23	16 (55.2)	27 (39.1)	
Duration of rheumatoid arthritis, months	103.9 ± 87.8	111.0 ± 69.2	0.666
**Rheumatoid factor**			0.329
Positive	20 (69.0)	54 (78.3)	
Negative	9 (31.0)	15 (21.7)	
**ACPA**			0.177
Positive	17 (34.6)	49 (79.0)	
Negative	9 (65.4)	13 (21.0)	
**Concomitant drug**			
Hydroxychloroquine			0.902
Yes	16 (55.2)	39 (56.5)	
No	13 (44.8)	30 (43.5)	
Leflunomide			0.665
Yes	10 (34.5)	27 (39.1)	
No	19 (65.5)	42 (60.9)	
Methotrexate			0.319
Yes	19 (65.5)	52 (75.4)	
No	10 (34.5)	17 (24.6)	
Sulfasalazine			0.111
Yes	7 (24.1)	7 (10.1)	
No	22 (75.9)	62 (89.9)	
Tacrolimus			0.986
Yes	5 (17.2)	12 (17.4)	
No	24 (82.8)	57 (82.6)	
**Comorbidity**			
Diabetes			0.669
Yes	1 (3.6)	5 (7.2)	
No	27 (96.4)	64 (92.8)	
Dyslipidemia			1.000
Yes	3 (10.7)	9 (13.0)	
No	25 (89.3)	60 (87.0)	
Hypertension			0.060
Yes	1 (3.6)	14 (20.3)	
No	27 (96.4)	55 (79.7)	
Osteoporosis			0.725
Yes	4 (14.3)	7 (10.1)	
No	24 (85.7)	62 (89.9)	
Vitamin D deficiency			0.272
Yes	1 (3.6)	9 (13.0)	
No	27 (96.4)	60 (87.0)	
**Baseline DAS28 with its subcomponents**			
DAS28	5.8 ± 1.2	5.8 ± 1.1	0.696
Tender joint count 28	9.6 ± 8.3	10.7 ± 74.3	0.782
Swollen joint count 28	6.6 ± 7.1	7.5 ± 5.7	0.872
Global health	55.3 ± 18.5	62.4 ± 20.2	0.167
ESR	54.7 ± 29.5	49.5 ± 27.3	0.379
CRP	3.0 ± 4.4	2.1 ± 2.5	0.113

BMI: body mass index; ACPA: anticyclic citrullinated peptide antibody; DAS28: disease activity score 28 joints; ESR: erythrocyte sedimentation rate; CRP: C-reactive protein.

As shown in Table 2, statistically significant associations between genotypes and RA remission were found for both TLR9 SNPs: T-allele carriers of rs352139 and rs352140 experienced approximately 3.3 and 4.5 times more frequent remission than patients with the CC genotype, respectively. A table of SNP with three genotypes is provided in the Supplementary section (Supplementary Table S2).

**Table 2:** Genotype association with the remission at 6 months treatment of TNF inhibitors.

Gene, rs number	Remission (DAS28 < 2.6)	No remission (DAS28 ≥ 2.6)	*p-*value
**TLR4 rs11536889**			0.578
GG, CG	11 (39.3)	23 (33.3)	
CC	17 (60.7)	46 (66.7)	
**TLR4 rs1927907**			1.000
CC, CT	27 (93.1)	64 (92.8)	
TT	2 (6.9)	5 (7.2)	
**TLR4 rs1927911**			0.178
AA, AG	21 (72.4)	40 (58.0)	
GG	8 (27.6)	29 (42.0)	
**TLR4 rs2149356**			0.335
TT, TG	21 (72.4)	56 (81.2)	
GG	8 (27.6)	13 (18.8)	
**TLR9 rs352139**			0.017
TT, CT	25 (89.3)	45 (65.2)	
CC	3 (10.7)	24 (34.8)	
**TLR9 rs352140**			0.008
TT, CT	26 (92.9)	46 (66.7)	
CC	2 (7.1)	23 (33.3)	

TLR: toll-like receptor; TNF-α: tumor necrosis factor-α.

Multivariable analysis (Table 3) included sex, age, and factors with *p* < 0.05 from the univariate analysis. Because significant linkage disequilibrium was observed between rs352139 and rs352140 (*r*^2^ = 0.95), only rs352139 was included in the multivariable analysis. Among the included factors, rs352139 was significantly associated with RA remission (95% CI 1.325–19.231, *p* = 0.018). After adjusting for related covariates, the remission rate in T-allele carriers of rs352139 was about 5.1 times that in patients with the CC genotype. The Hosmer–Lemeshow test showed that the fitness of the multivariable analysis model was satisfactory (χ^2^ = 0.907, 2 degrees of freedom, *p* = 0.636).

**Table 3:** Multivariate analysis to identify predictors for the remission rate at 6 months treatment of TNF inhibitors.

	Crude OR (95% CI)	*p-*value	Adjusted OR (95% CI)	*p-*value
Age < 65	2.618 (0.699–9.803)	0.153		
Male	2.653 (0.944–7.462)	0.064		
TLR9 rs352139^a^	4.444 (1.217–16.129)	0.024	5.05 (1.325–19.231)	0.018

Adjusted for age, sex, and TLR9 rs352139.

OR: odds ratio; CI: confidence interval.

^a^T carriers of rs352139.

**Figure 1:**
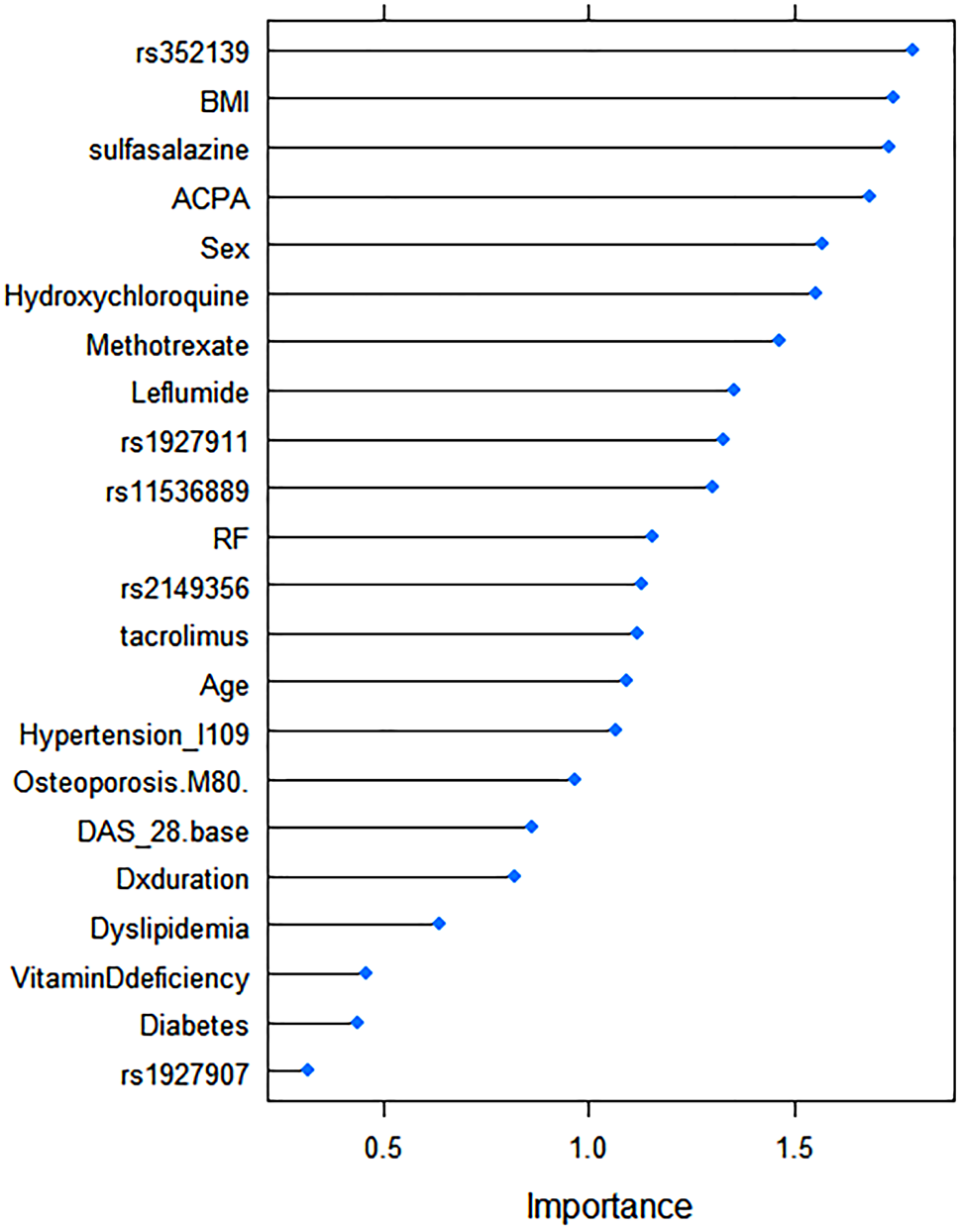
Variable importance using random forest to predict remission in patients with RA receiving TNF-α inhibitors. Figure was drawn using caret R package version 6.0-88 (https://github.com/topepo/caret/).

**Figure 2:**
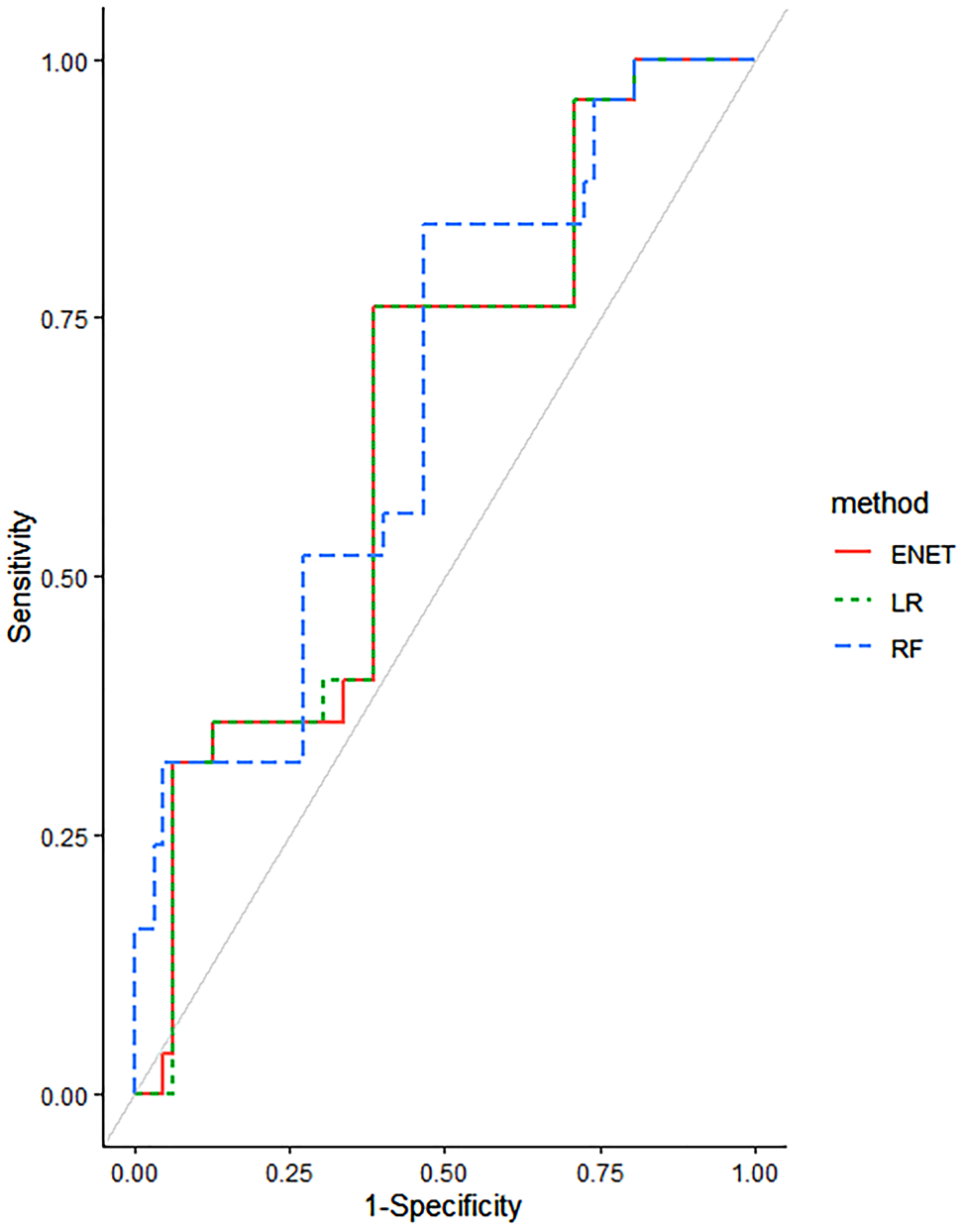
The receiver operating characteristic curves for predictive performance of elastic net (ENET), logistic regression (LR), and random forest (RF) models. Figure was drawn using caret R package version 6.0-88 (https://github.com/topepo/caret/).

As shown in Fig. 1, after feature selection using performing five-fold cross-validated random forest approach, four important variables from feature selection (rs352139, body mass index (BMI), sulfasalazine, and anti-citrullinated protein/peptide antibody (AC-PA)) were included in machine learning models. After performing five-fold cross-validated multivariate logistic regression, elastic net, random forest, support vector machine (SVM) models, the average area under the receiver-operating curve (AUROC), values across 100 random iterations were shown in Table 4. The AUROC values for multivariate logistic regression, elastic net, and random forest indicated good performances of the models; 0.71, 0.71, and 0.70 respectively (95% CI 0.594–0.827 for multivariate logistic regression and elastic models and 0.584–0.821, respectively). Linear kernel SVM and radial kernel SVM revealed sub-optimal performances of the models; AUROC values of 0.60 and 0.67, respectively (95% CI 0.416–0.782 and 0.53–0.813, respectively). Figure 2 showed AUROC curves of three models that exhibit good interpretability and prediction rate. Details for the packages used and parameters used for training models are provided in the Supplementary section (Supplementary Table S1).

**Table 4:** Comparisons of AUC for logistic regression, elastic net, random forest, and SVM models.

	AUROC	95% CI
Logistic regression	0.71	0.594–0.827
Elastic net	0.71	0.594–0.827
Random forest	0.70	0.584–0.821
SVM (linear)	0.60	0.416–0.782
SVM (radial)	0.67	0.530–0.813

AUROC: area under the receiver-operating curve; CI: confidence interval; SVM: Support vector machine.

## Discussion

The main finding of this study is that rs352139 of TLR9 was associated with treatment response to TNF-α inhibitors in RA patients. The remission rate in T-allele carriers of rs352139 was about 5 times that in patients with the CC genotype. Multivariate logistic regression and elastic net were proven to be the most suitable method in predicting remission in patients with RA, with AUROC values of 0.71 (95% CI 0.594–0.827 for both models).

TNF-α is a pro-inflammatory cytokine involved in the innate immune response^30^. It is involved in the pathogenesis of several inflammatory conditions, especially RA. As the TNF-α level is elevated in patients with RA, TNF-α inhibitors have been frequently used to treat of RA. Unlike other agents for RA therapy, TNF-α inhibitors target cytokines and are used to treat patients with advanced RA.

Damage-associated molecular patterns (DAMPs) are endogenous danger molecules that activate the innate immune system by interacting with TLRs. This evokes innate immune responses, including induction of inflammatory cytokines^31^. DAMPs play an important role in the initiation of inflammation during tissue injury without infection and are may also be involved in chronic inflammation including autoimmune diseases^12^. Once DAMPs are released during tissue injury, TLRs are activated, and the inflammatory cycle is initiated. The binding of TLRs to DAMPS activates the receptors, up-regulating pro-inflammatory mediators including cytokines and resulting in various inflammatory conditions and chronic inflammation^12^.

Ligand-bound TLRs interact with elements on the surface of pathogens and activate the MyD88-related pathways^31^, resulting in NFkB activation and cytokine gene expression^10^. This ultimately leads to the induction of molecules associated with inflammation and release of pro-inflammatory components such as TNF-α^32^. TLRs are known as inducers of TNF-α transcription^13^. Several studies have shown an increased expression of TLR4 on RA synovial fluid macrophages and RA synovial fibroblasts^33, 34^ and of TLR9 in RA synovial tissue fibroblasts and RA peripheral blood monocytes^18, 35^.

Our results revealed that TLR9 polymorphism was associated with the remission rate of RA patients taking TNF-α inhibitors. The T-allele carriers of rs352139 had a significantly higher remission rate than patients with the CC genotype. TLR9 is expressed by B cells and functions with the B cell receptor complex, resulting in the release of rheumatoid factor^36^. Previously, Bank et al.^22^ have reported an association of the GG genotype of rs352139 with nonresponse to TNF-α inhibitors in inflammatory bowel disease patients, which is in line with our findings. This association is possibly attributable to alteration in TLR function; however, further research is required to validate our results, as there are no published mechanistic studies on the association between this polymorphism and TNF-α inhibition or treatment response in advanced RA patients.

The utilization of machine learning approaches to predict remission in patients with RA receiving TNF-α inhibitors is novel in clinical research. In clinical settings, these models can be helpful in decision-making process. To overcome over-fitting, this study utilized random forest, an ensemble method of bootstrap aggregated binary classification trees^37^ for feature selection. We also demonstrated that multivariate logistic regression and elastic net, a penalized linear regression model that combine penalties of the lasso and ridge methods^38^, models outperformed other models. Hence, these models may be useful in predicting remission in patients on TNF-α inhibitor treatment.

The limitations of our study are its small sample size and the lack of a detailed mechanism. Nevertheless, to our knowledge, this is the first study to investigate the effects of genetic variations in the TLR4 and TLR9 genes on favorable response rates to RA treatment in patients taking TNF-α inhibitors. Moreover, this study provides important features and prediction models based on machine learning algorithms including logistic regression, elastic net, random forest and SVM for remission in patients with RA receiving TNF-α inhibitors. Since our study developed prediction models using TLR4 and TLR9 gene polymorphisms for remission of RA in patients taking TNF-α inhibitors, result of this study could be utilized to develop and design individually tailored TNF-α inhibitor treatments for RA patients.

## Supplementary Information

Supplementary Information.
