# Supplementary material for: Association of TLR 9 gene polymorphisms with remission in patients with rheumatoid arthritis receiving TNF-α inhibitors and development of machine learning models
Source: Sci Rep. 2021 Oct 11;11:20169. doi: 10.1038/s41598-021-99625-x (PMC8505487; doi:10.1038/s41598-021-99625-x)
Supplement: Supplementary file 1 — Supplementary Information. [file 41598_2021_99625_MOESM1_ESM.docx]

**Supplementary Table S1. Machine learning model specifics**

| Method | Hyperparameter | |
| --- | --- | --- |
|  | Model specification and search grids | Selected values |
| Elastic net | $\lambda$: 100 equally spaced values in logarithmic scale between 10^-4^ and 0  $\alpha$: 0, 0.2, 0.4, 0.6, 0.8, 1 | $\lambda$: 0.02205131  $\alpha$: 0 |
| Random forests | mtry: 1, 2, 3, 4, 5, 6, 7, 8, 9 | mtry: 8 |
| SVM with linear kernel | C: 0, 0.001, 0.005, 0.01, 0.05, 0.1, 0.25, 0.5, 0.75, 1, 1.25, 1.5, 1.75, 2, 5 | C: 1.5 |
| SVM with radial kernel | Sigma: 2^-15^, 2^-13^, 2^-11^, 2^-9^, 2^-7^, 2^-5^, 2^-3^, 2^-1^, 2, 2^3^  C: 2^-5^, 2^-3^, 2^-1^, 2, 2^3^, 2^5^, 2^7^, 2^9^, 2^11^, 2^13^, 2^15^ | Sigma: 0.03125  C: 2048 |

**Supplementary 1. Genotyping method**

Genotyping was performed using a single-base primer extension assay with TaqMan genotyping assay in a real-time PCR system (QuantStudio 3 Real-Time PCR, ABI) (Applied Biosystems, Carlsbad, CA, USA). Six SNPs – TLR4 (rs11536889, rs1927907, rs1927911, and rs2149356) and TLR9 (rs352139 and rs352140) – were screened by TaqMan allelic discrimination technique using RT-PCR. PCR was run in eight optical strips with a total volume of 25 μL comprised of 11.25 μL of DNA sample preparation and 13.75 μL of PCR reaction mix. The PCR reaction mix was a combination of 12.5 μL TaqMan Genotyping Master Mix and 1.25 μL of 40xTaqMan SNP Genotyping Assay Mix (Applied Biosystems). The Genotyping Master Mix consisted of AmpliTaq Gold DNA polymerase, deoxiribonucleotide triphosphates (dNTPs), and ROX passive reference and buffer components. The Genotyping Assay Mix was composed of forward and reverse primers for amplifying the sequence of interest and two TaqMan MGB probes (tagged with VIC/FAM reporter dyes) to detect specific SNP alleles. After 10 minutes of denaturation at 95 °C, PCR was run for 40 cycles at 92 °C for 15 seconds and at 60 °C for 60 seconds. During the PCR cycle, primers were located at a conserved part of the 5’ non-coding region on the gene, while probes were bound to their complementary sequence.

**Supplementary Table S2. Genotype association with the remission at 6 months treatment of TNF inhibitors**

| Gene, rs number | Remission  (DAS28 < 2.6) | No remission  (DAS28 ≥ 2.6) | *p-*value |
| --- | --- | --- | --- |
| TLR4 rs11536889 |  |  | 0.382 |
| GG | 2 (7.1) | 1 (1.4) |  |
| CG | 9 (32.1) | 22 (31.9) |  |
| CC | 17 (60.7) | 46 (66.7) |  |
| TLR4 rs1927907 |  |  | 1.000 |
| CC | 15 (51.7) | 37 (53.6) |  |
| CT | 12 (41.4) | 27 (39.1) |  |
| TT | 2 (6.9) | 5 (7.2) |  |
| TLR4 rs1927911 |  |  | 0.381 |
| AA | 8 (27.6) | 13 (18.8) |  |
| AG | 13 (44.8) | 27 (39.1) |  |
| GG | 8 (27.6) | 29 (42.0) |  |
| TLR4 rs2149356 |  |  | 0.535 |
| TT | 9 (31.0) | 29 (42.0) |  |
| TG | 12 (41.4) | 27 (39.1) |  |
| GG | 8 (27.6) | 13 (18.8) |  |
| TLR9 rs352139 |  |  | 0.044 |
| TT | 8 (28.6) | 11 (15.9) |  |
| CT | 17 (60.7) | 34 (49.3) |  |
| CC | 3 (10.7) | 24 (34.8) |  |
| TLR9 rs352140 |  |  | 0.025 |
| TT | 8 (28.6)) | 12 (17.4) |  |
| CT | 18 (64.3) | 34 (49.3) |  |
| CC | 2 (7.1) | 23 (33.3) |  |

TLR: toll-like receptor; TNF-α: tumor necrosis factor-α
